# News and Notes

**Published:** 1907-06

**Authors:** 


### NEWS AND NOTES.

Thirty cases of trachoma are reported among Russian and Japanese immigrants at Fresno.

A lively controversy is now waging in the London medical and lay press regarding the use of alcoholic beverages.

A bill, preventing the practice of Christian Science in Delaware, has been signed by Gov. Lea of that State.

Prof. William L. Richardson, dean of Harvard Medical School, has tendered his resignation, which has been accepted.

It is reported that Dr. T. F. Brewster is to resign his position as superintendent of Grady Hospital after fourteen years' service, on account of ill health.

Dr. George M. Whitaker, inspector dairy department of agriculture, of Washington, D. C., delivered a lecture at the Carnegie Library on the "Model Dairy Farm."

Professor A. R. Mosetig-Moorhof died in Vienna April 26th. He was for years assistant professor of surgery at the University of Viennt, his alma mater. 1886 he introduced idofor minto surgery.

The University of Maryland conferred the honorary degree of LL.D. on one of its alumni, Major James S. Carroll, United States Army, at the centennial celebration held in Baltimore May 30 to June 2.

The Board of Regents of the University of Nebraska has recommended that the degree of Doctor of Laws be conferred on Major James Carroll, surgeon, U. S. Army, at the annual commencement exercises in June.

Dr. John S. Fulton, for ten years secretary of the State Board of Health, of Baltimore, has resigned to become secretary of the International Conference on Tuberculosis. He will make his headquarters at Washington, D. C.

---

A plan to hold an international conference on opium is attracting a good deal of attention, and it seems quite probable that such a conference will be held and that the United States, China, Japan, England, Holland, Germany, and France will participate.

---

The Medical Society of the County of New York has sent a letter to its members asking them to use personal influence with their representatives in order to secure the defeat of a bill regulating the practice of optometry which makes partial doctors of opticians.

---

Prof. A. Politzer will resign his position as head of the Vienna ear clinic this summer on account of his age. He will also cease his lectures after 46 years of service. A formal celebration has been planned, but on account of a number of deaths in his immediate family he requested that no public celebration be given.

---

Among the appropriations recently voted by the German parliament is $50,000 for repression of typhoid fever and $30,000 for tuberculosis; $16,000 for study of sleeping sickness; $17,500 for the approaching International Congress for Hygiene at Berlin, September 23-29; $25,000 for research on syphilis; $6,500 for investigation of the statistics of accidents and $10,000 for combating infant mortality.

---

At the Savannah meeting of the Medical Association of Georgia the following officers were elected for the ensuing year: President, Dr. M. A. Clark, Macon; vice-presidents, Drs. Ralph M. Thompson, Savannah, and E. E. Murphy, Augusta; secretary-treasurer, Dr. Louis H. Jones, Atlanta; delegates to American Medical Association, Drs. George R. White, Savannah, and F. H. Harris, Atlanta.

Fitzgerald was selected as the place for holding the next meeting.

